# Association between Mediterranean Diet and Fatty Liver in Women with Overweight and Obesity

**DOI:** 10.3390/nu14183771

**Published:** 2022-09-13

**Authors:** Alessandro Leone, Simona Bertoli, Giorgio Bedogni, Laila Vignati, Marta Pellizzari, Alberto Battezzati

**Affiliations:** 1 International Center for the Assessment of Nutritional Status (ICANS), Department of Food, Environmental and Nutritional Sciences (DeFENS), University of Milan, 20133 Milan, Italy; 2 Laboratory of Nutrition and Obesity Research, Istituto Auxologico Italiano, IRCCS, 20145 Milan, Italy; 3 Department of Medical and Surgical Sciences, Alma Mater Studiorum-University of Bologna, 40126 Bologna, Italy; 4 Internal Medicine Unit Addressed to Frailty and Aging, Department of Primary Health Care, S. Maria delle Croci Hospital, AUSL Romagna, 48121 Ravenna, Italy

**Keywords:** Mediterranean diet, non-alcoholic fatty liver disease, liver steatosis, overweight, obesity

## Abstract

Obesity is a risk factor for NAFLD. However, not all people with obesity have an excessive intrahepatic fat content. Adherence to a high-quality dietary pattern may also promote liver health in obesity. A cross-sectional study of 2967 women with overweight and obesity was carried out to assess the association between a Mediterranean diet and fatty liver. All women underwent clinical examination, anthropometric measurements, blood sampling, ultrasound measurements of abdominal visceral and subcutaneous fat, and assessment of adherence to the Mediterranean diet using the 14-item MEDAS questionnaire. Fatty liver index (FLI), NAFLD fatty liver steatosis (NAFLD-FLS) and hepatic steatosis index (HSI) were calculated. In women with obesity, the MEDAS score was inversely associated with FLI (β = −0.60, 95% CI: −1.04, −0.16, *p* = 0.008), NAFLD-FLS (β = −0.092, 95% CI: −0.134, −0.049, *p* < 0.001) and HSI (β = −0.17, 95% CI: −0.30, −0.04, *p* = 0.011). Stronger associations were observed in premenopausal women with obesity. Mediterranean diet was inversely associated with NAFLD-FLS in women with overweight, independently of menopausal status. In conclusion, Mediterranean diet is associated with a better liver status in women with overweight and obesity. This may have a public health impact and be useful in drafting nutritional guidelines for NAFLD.

## 1. Introduction

Non-alcoholic fatty liver disease (NAFLD) is a condition resulting from excessive lipid accumulation in the liver (triglyceride content >5% of organ weight) in individuals with low alcohol consumption (<20 g/day in women and <30 g/day in men) [1]. NAFLD encompasses a spectrum of conditions, including simple steatosis, non-alcoholic steatohepatitis (NASH) and cirrhosis, which can sometimes progress to hepatocarcinoma [1]. The reference method for diagnosing NAFLD is liver biopsy [2]. However, this is an expensive and invasive procedure that can lead to clinical complications in 1% of patients and death in 0.01% of them [3]. Non-invasive fatty liver indices are easy-to-use diagnostic tools with good accuracy in detecting NAFLD, and because of this fact, several European scientific societies recommend their use for the diagnosis and management of NAFLD, especially when imaging techniques are not available or not feasible [4]. In Western countries, NAFLD affects 20–30% of the adult general population [5], with a prevalence higher in men than in women in the reproductive age. However, after menopause, NAFLD occurs more frequently in women [6]. 

Obesity is an established risk factor for the development of NAFLD, and 50% to 75% of people with obesity also have NAFLD [7]. Excess body weight is associated with excess visceral fat accumulation and insulin resistance, which are the two main actors in the pathogenesis of NAFLD. However, NAFLD is not inextricably linked to obesity; some individuals with obesity have normal liver fat, while others with normal weight have NAFLD [8]. Environmental factors, such as adherence to a high-quality dietary pattern, may favorably influence the metabolic profile of individuals with overweight and obesity, preventing or delaying the development of NAFLD.

The Mediterranean diet is a high-quality dietary pattern characterized by high consumption of fruits and vegetables, cereals, especially whole grains, nuts and legumes, with olive oil as the main source of added fats, along with high to moderate intake of fishery products, moderate consumption of dairy products, white meat and eggs, low consumption of red or processed meat and moderate intake of alcohol (especially wine at meals) [9]. The Mediterranean diet is inversely associated with obesity, metabolic syndrome, cardiovascular disease (CVD) and type 2 diabetes [10]. Some cohort studies suggest that Mediterranean diet may be beneficial for the primary prevention of NAFLD in the general population [11,12]. An inverse association between adherence to the Mediterranean diet and risk of NAFLD has also been reported by a number of case–control [13,14] and cross-sectional [15,16,17] studies of healthy subjects and convenience samples of subjects with selected metabolic disease. However, we are not aware of studies performed in patients with excess body weight. Studies investigating the association between Mediterranean diet and non-invasive indices of fatty liver in these patients are, therefore, required. Knowledge of the relationship between diet and fatty liver is necessary to develop effective strategies for the prevention and treatment of NAFLD associated with obesity [18]. Previously, several randomized controlled trials have shown the potential benefits of the Mediterranean diet against the progression of hepatic steatosis in individuals with obesity [19,20,21,22]. Moreover, it has been reported that the Mediterranean diet is associated with lower visceral fat accumulation [23], greater insulin sensitivity [24] and fewer metabolic alterations in individuals with obesity [25]. In light of this evidence, it is presumable that the Mediterranean diet is also associated with lower indices of fatty liver in individuals with excess body weight. In women, however, the association between diet and liver health could be influenced by menopausal status. With the advent of menopause, in fact, a redistribution of adipose tissue from the gluteo-femoral to the abdominal region occurs, increasing cardiometabolic risk. To verify these assumptions, the aim of this study was to evaluate the association between Mediterranean diet and non-invasive indices of fatty liver in a large sample of women with overweight and obesity.

## 2. Materials and Methods

### 2.1. Study Design and Protocol

We performed a cross-sectional study of 2967 consecutive women with overweight and obesity referring to the International Center for Nutritional Status Assessment (ICANS, University of Milan, Milan, Italy) from September 2010 to February 2020, to devise a nutritional assessment and obtain a personalized dietary intervention to lose weight. The study was conducted following the guidelines established by the Declaration of Helsinki. The Ethics Committee of the University of Milan approved the study procedures (study protocol No. 23/2016). Patients gave written informed consent for the use of their data for research purposes.

The inclusion criteria were: women, age ≥ 18 years, BMI ≥ 25 kg/m^2^ and alcohol intake ≤ 20 g/day. The exclusion criteria were: diabetes, cardiovascular disease, cancer within the past 5 years, hepatitis C virus infection, hepatitis B virus infection, liver cirrhosis, neurological disease, gastrointestinal disease, cardiac failure, renal failure, pulmonary insufficiency and use of drugs known to cause lipodystrophy. 

The patients underwent a clinical history and a physical examination. An appointed physician collected the data on the personal and family occurrence of CVD, diabetes and other chronic disease, previous and current pharmacological treatment, menopausal status, blood pressure and lifestyle habits, such as smoking and structured physical activity. Blood pressure was measured using international guidelines. Such guidelines changed during the study period but not with regard to the method of measurement of blood pressure. Women reporting absence of menstrual cycle for at least 12 months were defined as postmenopausal.

A fasting blood sample was drawn between 08:30 and 09:00 AM for the measurement of blood glucose, insulin, triglycerides, total-, low-density-lipoprotein (LDL)-, high-density-lipoprotein (HDL)-cholesterol and alanine transaminase (ALT), aspartate transaminase (AST) and gamma-glutamyl-transferase (GGT).

Thereafter, an experienced physician measured the thicknesses of visceral adipose tissue (VAT) and subcutaneous adipose tissue (SAT) at the abdominal level using ultrasonography (Logiq 3 Pro, GE Healthcare, Boston, MA, USA) with a 7.5 MHz linear probe and a 3.5 MHz convex-array probe. The measurements, recorded at the end of expiration, were taken 1 cm above the umbilicus. VAT was defined as the distance between the posterior surface of the rectus abdominis muscle and the anterior wall of the aorta. SAT was defined as the distance between the external face of the rectus abdominis muscle and the epidermis [26,27]. 

Lastly, a registered dietitian took anthropometric measurements following international guidelines [28]. Weight was measured with an electronic scale with an accuracy of 100 g (Seca 700, Seca Corporation, Hamburg, Germany). Height was measured with a vertical stadiometer with an accuracy of 0.1 cm. Waist circumference (WC) was measured with an accuracy of 0.5 cm using a nonelastic tape placed at the midpoint between the last rib and the iliac crest. The thicknesses of four skinfolds (biceps, triceps, subscapular and suprailiac) were measured using a skinfold caliper (Holtan Ltd., Crymych, Wales). Fat mass was estimated from the Durnin–Womersley equation [29]. Percent fat mass (%FM) was calculated as (fat mass (kg)/body weight (kg)) × 100.

Metabolic syndrome was diagnosed according to the harmonized definition [30].

### 2.2. Outcome Assessment

Fatty liver status was evaluated using the following non-invasive indices:

- Fatty liver index (FLI) [31] = e^LP^/(1 + e^LP^) × 100 where LP (linear predictor) = 0.953 × ln(triglycerides (mg/dL)) + 0.139 × BMI (kg/m^2^) + 0.718 × ln (GGT (U/L)) + 0.053 × WC (cm) − 15.745;
- Non-alcoholic fatty liver disease–fatty liver steatosis (NAFLD-FLS) [32] = −2.89 + 1.18 × Metabolic syndrome (0 = No; 1 = Yes) + 0.45 × type 2 diabetes mellitus (2 = Yes; 0 = No) + 0.15 × insulin (mU/L) + 0.04 × AST (U/L) − 0.94 × AST (U/L)/ALT (U/L);
- Hepatic steatosis index (HSI) [33] = 8 × (ALT (U/L)/AST (U/L)) + BMI (kg/m^2^) + 2 if woman + 2 if diabetes mellitus.

### 2.3. Mediterranean Diet Adherence

Adherence to the Mediterranean diet was evaluated using the 14-item Mediterranean diet adherence screener (MEDAS), a short questionnaire developed and validated in the PREDIMED trial [34]. Such questionnaire investigates food preferences and the frequency of consumption of foods typical of the Mediterranean diet and of Western diets. For each item, one point is assigned if the Mediterranean criterion is met, as reported in Supplementary Table S1 [35,36,37]. The score of MEDAS ranges from 0 to 14 points.

### 2.4. Statistical Analysis

As most continuous variables were not normally distributed, all are presented as median and interquartile range. Discrete variables are presented as frequency and percentage. Linear regression models were used to evaluate the association between the MEDAS score (continuous) and fatty liver indices. Age (continuous), BMI class (discrete; 0 = overweight, 1 = obesity), %FM (continuous), VAT (continuous), SAT (continuous), total cholesterol (continuous), HDL (continuous), glucose (continuous), menopausal status (discrete, 0 = premenopausal, 1 = postmenopausal), smoking (discrete; 0 = non-smoker, 1 = ex-smoker, 2 = smoker), physical activity (discrete; 0 = no, 1 = at least 2 h/week), statins (discrete, 0 = no, 1 = yes) were included as potential confounders. To evaluate the association of the Mediterranean diet with fatty liver, a BMI × MEDAS (discrete x continuous) interaction was added to the models; because the BMI × MEDAS interaction was significant for all models, we reported the results separately for each BMI class. No evidence of multicollinearity was found among the predictors. Multivariable fractional polynomials were used to model non-linear associations of continuous predictors with the outcome. Because we found some violations of the homoscedasticity assumption in some regression models, robust confidence intervals were calculated for all models [23]. A *p* value of <0.05 was considered statistically significant. Statistical analysis was performed using Stata 12.0 (Stata Corporation, College Station, TX, USA).

## 3. Results

The measurements of the patients stratified by BMI category are reported in Table 1.

Table 2 reports the association of the Mediterranean diet score with fatty liver indices.

**Figure 1:**
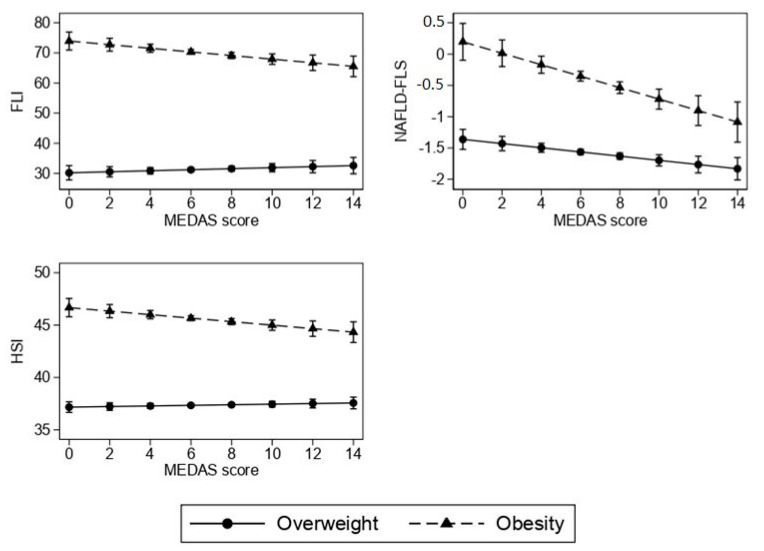
Association of Mediterranean diet (MEDAS score) with indices of fatty liver according to BMI category. Values are marginal means and 95% confidence intervals estimated from multivariable linear regression.

MEDAS was associated with the indices of fatty liver, but the magnitude of the association differed among the BMI classes. In women with obesity, a 1-point increase in MEDAS was associated with a 0.60 reduction in FLI (95% CI: −1.04; −0.16, *p* = 0.008), a 0.092 reduction in NAFLD-FLS (95% CI: −0.134; −0.049, *p* < 0.001) and a 0.17 reduction in HSI (95% CI: −0.30; −0.04, *p* = 0.011). In women with overweight, a 1-point increase in MEDAS was associated with a 0.034 reduction in NAFLD-FLS (95% CI: −0.057; −0.010, *p* = 0.005), while no association between the MEDAS score and FLI and HSI was found. Figure 1 shows the marginal mean of indices of fatty liver associated with the MEDAS score in women with overweight and obesity.

Given the impact of menopause on body composition and metabolism, we also stratified the analysis for the menopausal status (Table 3).

**Figure 2:**
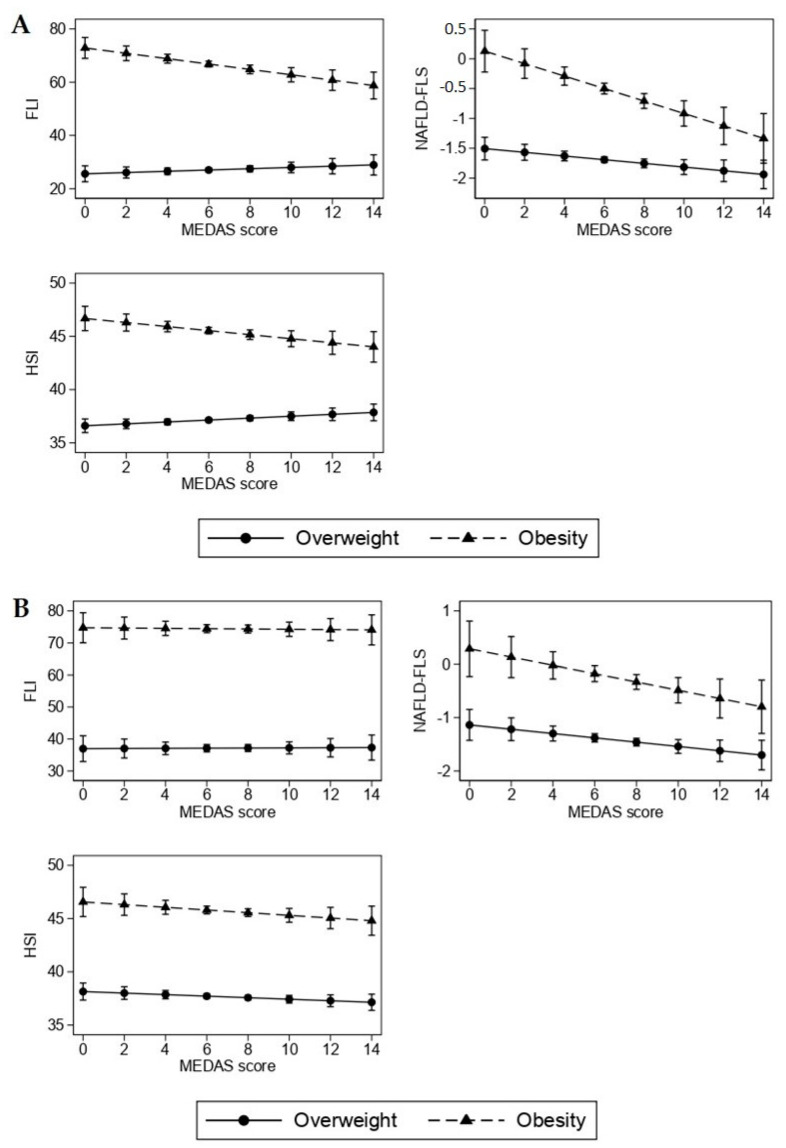
Association of Mediterranean diet (MEDAS score) with indices of fatty liver according to BMI category in premenopausal (**A**) and postmenopausal (**B**) women.

In premenopausal women with obesity, MEDAS was inversely associated with all indices of fatty liver, while in postmenopausal women, MEDAS was inversely associated only with NAFLD-FLS (−0.077, 95% CI: −0.148; −0.007, *p* = 0.032, for 1-unit increase). In women with overweight, MEDAS was stably associated with NAFLD-FLS, independently of menopausal status. Figure 2 shows the marginal means of indices of fatty liver associated with MEDAS in pre- and postmenopausal women with overweight and obesity.

## 4. Discussion

Non-invasive fatty liver indices are commonly used in clinical practice as simple and accurate tools to screen for NAFLD, especially when imaging techniques are not available. Among them, FLI and NAFLD-FLS have been extensively validated in external populations [4]. Recently, HSI has also been externally validated and has been found to perform similarly to the FLI in predicting NAFLD [38]. In the present study, higher adherence to the Mediterranean diet was associated with lower indices of fatty liver in women with overweight and obesity, suggesting that diet quality, even without energy restriction, might promote liver health despite excess body weight. These findings are consistent with those of trials showing that the Mediterranean diet lowers liver fat in people with obesity, even without energy restriction and weight loss [21]. Therefore, choosing a Mediterranean diet as dietary intervention to achieve weight loss might improve liver health, even if it fails to improve weight status. Taken together, these findings may have relevance for public health and may be useful to draft nutritional guidelines aimed at the prevention and management of NAFLD.

The exact pathophysiological pathway leading to fat accumulation in the liver is not entirely clear [39]. However, the prevailing view is that it occurs as a result of an initial alteration of insulin sensitivity in adipose tissue, which would lead to uninhibited lipolysis, which, in turn, produces increased fatty acid (FA) flow to the liver and other organs (e.g., muscle) [40]. It is estimated that 60% of the triglycerides in the liver come from adipose tissue [41]. VAT is the main source of FA to the liver [42,43]. In subjects with obesity, VAT delivers 20% of total fatty acid intake to the liver, as compared to 5% in lean subjects [44]. Peripheral insulin resistance also enhances hepatic de novo lipogenesis [45,46], increasing hepatic triglyceride accumulation. This pathway accounts for 25% of hepatic triglyceride content [41,47]. This may, in turn, lead to dysregulation of VLDL production and secretion [48,49,50], which would be increased as a compensatory response to intrahepatocyte triglyceride accumulation, although this pathway seems to be insufficient to normalize the elevated hepatic triglyceride content [40]. The mechanisms proposed to explain the impact of Mediterranean diet on hepatic fat deposition involve the ability of diet to regulate general and visceral adiposity, as well as the specific biochemical processes occurring in the liver. In fact, it has been previously reported that there is an inverse association between adherence to the Mediterranean diet and the amount of abdominal visceral fat [23,51]. Moreover, as a result of higher consumption of fruits and vegetables and olive oil, individuals following the Mediterranean diet have higher intakes of dietary polyphenols and monounsaturated fatty acids, which have been shown to inhibit de novo lipogenesis and improve peripheral insulin sensitivity, mainly through their antioxidant, anti-inflammatory and antifibrotic effects [52,53,54,55]. The Mediterranean diet is also characterized by high consumption of fish and seafood, which contributes to increased intake of omega-3 PUFAs, which have been shown to reduce hepatic lipid accumulation and improve liver enzymes and insulin sensitivity, as well as having anti-inflammatory effects. In contrast, low omega-3 PUFAs intake has been correlated with the development of NAFLD and progression to NASH and cirrhosis [56,57,58]. In addition, the high fiber content of the Mediterranean diet may positively influence body weight and the composition of the gut microbiota [59] by increasing the levels of bacteria that produce short-chain fatty acids, such as butyrate and propionate, which have been shown to have insulin-sensitizing effects [60,61]. The Mediterranean diet is also low in saturated fats, which have been shown to enhance de novo lipogenesis, insulin resistance and increased hepatic triglyceride content [62,63,64]. On the other hand, women not following the Mediterranean diet may have a higher consumption of ultra-processed foods [65], whose consumption has been associated with overweight and obesity [66], visceral fat accumulation [67], insulin resistance, diabetes [68,69] and NAFLD [70].

Our results also show a stronger inverse association between Mediterranean diet and fatty liver indices in women with obesity than in women with overweight. Women with obesity had higher fatty liver indices than overweight women who had a medium–low risk of NAFLD, i.e., a median FLI < 30. Clinical trials have shown that the Mediterranean diet, in addition to reducing liver fat, improves insulin sensitivity and helps the reversion of metabolic syndrome, even without weight loss [21,71]. It follows that women at high risk of NAFLD may benefit more from increased adherence to the Mediterranean diet. Unlike the other fatty liver indices studied here, NAFLD-FLS does not include body weight and considers metabolic parameters, including insulin and metabolic syndrome. It is therefore possible that this index is more sensitive to the effect of diet quality, especially without weight loss. This may explain why NAFLD-FLS was inversely associated with Mediterranean diet adherence in both women with overweight and obesity, while such association was detected for FLI and HSI only in women with obesity. The same fact could explain the lack of association between FLI, HSI and Mediterranean diet in postmenopausal women. Menopause is accompanied by a physiological redistribution of adipose tissue, which tends to accumulate preferentially at the abdominal level. Therefore, the inclusion of parameters such as BMI and waist circumference could mitigate the association between fatty liver indices and adherence to the Mediterranean diet in postmenopausal women.

To the best of our knowledge, no study has assessed the association between adherence to the Mediterranean diet and fatty liver indices in women with overweight and obesity. In addition to its novelty, the present work has some strengths. First, the data are from a large population. Second, we used indices of fatty liver that have been externally validated and shown to have good and similar predictive abilities. Third, we controlled for a wide range of covariates, body composition and abdominal fat distribution, metabolic status and lifestyle, all variables that may impact metabolic status and risk of fatty liver. However, our study is not without limitations. First, a cross-sectional study design cannot prove a cause–effect relationship. Second, although we chose externally validated fatty liver indices, the use of such indices does not allow for the exact separation of individuals with NAFLD from those without. However, compared to liver biopsy, these indices are non-invasive, simple to use in clinical practice and cost effective. In addition, some of them, such as the FLI and NAFLD-FLS, are recommended by several European scientific societies for liver health screening in large epidemiological studies and when imaging techniques are not available [4]. Third, body composition was assessed using surrogate techniques, such as skinfold thickness measurement and ultrasonography. Body skinfolds are largely used in clinical practice but provide only an estimation of total body fat. Ultrasonography provides the thickness, not the area, of abdominal fat compartments. However, some studies show a good association between ultrasound measurements and areas measured by reference methods, such as computed tomography and magnetic resonance imaging [26,72]. Fourth, although it is valuable for its designed aim, the MEDAS questionnaire is a short dietary screener and therefore considers only the consumption of few specific foods [34]. Fifth, our sample included only Caucasian individuals, so these results need confirmation in individuals of other ethnicities.

## 5. Conclusions

In conclusion, the Mediterranean diet is inversely associated with indices of fatty liver in women with overweight and obesity. Women with obesity, especially during the premenopausal period, may benefit more from following a Mediterranean-style diet. Nevertheless, the Mediterranean diet could also improve liver health in overweight and postmenopausal women. These findings should be confirmed by randomized clinical trials using, at the very least, imaging techniques to diagnose NAFLD. 

## Figures and Tables

**Table 1:** Characteristics of patients.

	Total N = 2967			Overweight N = 1691			Obesity N = 1276		
	Median	P25	P75	Median	P25	P75	Median	P25	P75
Age (years)	47	39	55	47	38	54	48	39	56
BMI (kg/m^2^)	29.2	27.0	32.6	27.3	26.1	28.5	33.3	31.5	36.3
Body fat (%)	40.3	37.9	42.4	38.8	36.6	40.8	41.9	40.1	44.1
Waist circumference (cm)	96.0	90.0	103.3	91.5	87.0	95.5	104.5	99.0	110.5
VAT (cm)	4.5	3.4	6.2	3.9	3.0	5.0	5.8	4.3	7.5
SAT (cm)	3.0	2.3	3.7	2.7	2.1	3.2	3.5	2.8	4.3
Triglycerides (mg/dL)	87	65	120	81	60	110	98	72	134
Total cholesterol (mg/dL)	210	185	238	209	184	239	211	186	237
LDL cholesterol (mg/dL)	132	111	159	131	109	158	134	113	160
HDL cholesterol (mg/dL)	62	53	72	64	55	75	58	50	67
Serum glucose (mg/dL)	94	89	101	93	88	99	96	90	102
Insulin (U/L)	10.0	7.3	14	8.5	6.5	11.2	12.8	9.0	17.4
HOMA-IR	2.3	1.7	3.3	2.0	1.5	2.6	3.0	2.1	4.3
SBP (mm Hg)	120	110	130	120	110	125	122	120	130
DBP (mm Hg)	80	70	80	75	70	80	80	70	85
ALT (U/L)	18	14	24	16	13	22	19	15	27
AST (U/L)	18	15	21	17	15	21	18	15	22
ALT/AST	1.00	0.84	1.22	0.94	0.80	1.15	1.09	0.90	1.31
GGT (U/L)	16	12	24	15	11	21	19	13	26
FLI	44	25	71	28	18	42	74	56	87
NAFLD-FLS	−1.42	−2.07	−0.36	−1.80	−2.28	−1.11	−0.67	−1.57	0.46
HSI	40	37	44	37	35	39	45	42	48
Mediterranean score (MEDAS)	6	5	8	7	5	8	6	5	8
	N	%		N	%		N	%	
Menopausal status									
Premenopausal	1697	57.2		981	58		716	56.1	
Postmenopausal	1270	42.8		710	42		560	43.9	
Smoking									
Non-smoker	1623	54.7		899	53.2		724	56.7	
Ex-smoker	543	18.3		315	18.6		228	17.9	
Smoker	801	27		477	28.2		324	25.4	
Physically active (≥2 h/week)	711	24		457	27		254	19.9	
Statins	97	3.3		53	3.1		44	3.4	
Triglycerides ≥ 150 mg/dL or treatment	458	15.4		196	11.6		262	20.5	
HDL ≤ 50 mg/dL or treatment	575	19.4		245	14.5		330	25.9	
BP ≥ 130/85 mm Hg or treatment	1162	40.9		498	31		664	54	
Glucose 100–125 mg/dL	840	28.3		379	22.4		461	36.1	
Metabolic syndrome	724	24.4		252	14.9		472	37	

Abbreviations: VAT, visceral adipose tissue; SAT, subcutaneous adipose tissue; SBP, systolic blood pressure; DBP, diastolic blood pressure; ALT, alanine transaminase; AST, aspartate transaminase; GGT, gamma glutamyl-transferase; FLI, fatty liver index; NAFLD-FLS, NAFLD fatty liver score; HIS, hepatic steatosis index; BP, blood pressure.

**Table 2:** Association of Mediterranean diet with fatty liver indices in women with overweight and obesity.

	Overweight N = 1691			Obesity N = 1276		
	β	95% CI	*p* Value	β	95% CI	*p* Value
FLI	0.17	−0.18; 0.52	0.344	−0.60	−1.04; −0.16	0.008
NAFLD-FLS	−0.034	−0.057; −0.010	0.005	−0.092	−0.134; −0.049	<0.001
HSI	0.03	−0.05; 0.10	0.452	−0.17	−0.30; −0.04	0.011

Values are linear regression coefficients associated with one-point increase in the Mediterranean diet score (MEDAS). The underlying models are adjusted for age, BMI class, FM (%), VAT, SAT, total cholesterol, HDL cholesterol, glucose, statins, menopausal status, smoking status and physical activity. Abbreviations: FLI, fatty liver index; NAFLD-FLS, NAFLD fatty liver steatosis; HSI, hepatic steatosis index.

**Table 3:** Association of Mediterranean diet with fatty liver indices in women affected by overweight and obesity according to menopausal status.

	Overweight			Obesity		
	N = 981			N = 716		
	β	95% CI	*p* Value	β	95% CI	*p* Value
Premenopausal						
FLI	0.24	−0.23; 0.71	0.323	−1.01	−1.63; −0.39	0.001
NAFLD-FLS	−0.031	−0.061; −0.001	0.042	−0.104	−0.158; −0.051	<0.001
HSI	0.09	−0.01; 0.19	0.075	−0.19	−0.37; −0.01	0.037
Postmenopausal						
FLI	0.02	−0.53; 0.58	0.931	−0.05	−0.70; 0.60	0.886
NAFLD-FLS	−0.040	−0.080; −0.001	0.045	−0.077	−0.148; −0.007	0.032
HSI	−0.07	−0.18; 0.04	0.193	−0.13	−0.32; 0.07	0.196

Values are linear regression coefficients associated with one-point increase in the Mediterranean score (MEDAS score). Model adjusted for age, BMI class, FM (%), VAT, SAT, total cholesterol, HDL cholesterol, glucose, statins, smoking and physical activity. Abbreviations: FLI, fatty liver index; NAFLD-FLS, NAFLD fatty liver steatosis; HSI, hepatic steatosis index.

## Data Availability

The data presented in this study are available on reasonable request from the corresponding author.

## Supplementary Materials

The following supporting information can be downloaded at: https://www.mdpi.com/article/10.3390/nu14183771/s1, Table S1: Questions and criteria for assessing the adherence to the Mediterranean diet.
