# Supplementary material for: Association between Mediterranean Diet and Fatty Liver in Women with Overweight and Obesity
Source: Nutrients. 2022 Sep 13;14(18):3771. doi: 10.3390/nu14183771 (PMC9501123; doi:10.3390/nu14183771)
Supplement: Supplementary file 1 [file nutrients-14-03771-s001.zip › nutrients-1858011-supplementary/TableS1.pdf]

Table S1: Questions and criteria for assessing the adherence to the Mediterranean diet

MEDAS Question	Criteria for 1 Point
1. Do you use olive oil as the principal source of fat for cooking?	Yes
2. How much olive oil do you consume per day (including that used in frying, salads, meals eaten away from home, etc.)?	≥4 tablespoons/day
3. How many servings of vegetables do you consume per day?	≥2 servings/day
4. How many pieces of fruit (including fresh-squeezed juice) do you consume per day?	≥3 servings/day
5. How many servings of red meat, hamburger, or sausages do you consume per day?	<1 serving/day
6. How many servings (12 g) of butter, margarine, or cream do you consume per day?	<1 serving/day
7. How many carbonated or sugar-sweetened beverages do you consume per day?	<1 serving/day
8. Do you drink wine? How much do you consume per week?	≥3 glasses/week
9. How many servings of pulses do you consume per week?	≥3 servings/week
10. How many servings of fish/seafood do you consume per week?	≥3 servings/week
11. How many times do you consume commercial (not homemade) pastry such as cookies or cake per week?	<3 times/week
12. How many times do you consume nuts per week?	≥1 servings/week
13. Do you prefer to eat chicken, turkey, or rabbit instead of beef, pork, hamburgers, or sausages?	Yes
14. How many times per week do you consume dishes prepared soffritto?	≥2 servings/week
